# Alternative Approaches to Feeding Small Ruminants and Their Potential Benefits

**DOI:** 10.3390/ani14060904

**Published:** 2024-03-14

**Authors:** Sofiane Boudalia, Samir Smeti, Mahilet Dawit, Ewonetu Kebede Senbeta, Yassine Gueroui, Vassilios Dotas, Aissam Bousbia, George K. Symeon

**Affiliations:** 1 Laboratory of Biology, Water and Environment, Faculty of Nature and Life and Earth Sciences and the Universes, 8 Mai 1945 University Guelma, BP 401, Guelma 24000, Algeria; gueroui.yassine@univ-guelma.dz (Y.G.); bousbia.aissam@univ-guelma.dz (A.B.); 2 Laboratory of Animal Production and Forages, National Institute of Agronomic Research of Tunis University of Carthage, Rue Hédi Karray, Ariana 2049, Tunisia; sam_fsb@live.fr; 3 School of Animal and Range Science, College of Agriculture and Environmental Science, Haramaya University, Dire Dawa P.O. Box 138, Ethiopia; mahidawit@gmail.com; 4 Department of Animal Production Studies, College of Veterinary Medicine and Agriculture, Addis Ababa University, Addis Ababa, Ethiopia; ewonetu2011@gmail.com; 5 Department of Animal Production, Faculty of Agriculture, Aristotle University of Thessaloniki, 54124 Thessaloniki, Greece; vdotas@agro.auth.gr; 6 Research Institute of Animal Science, HAO-Demeter, 58100 Giannitsa, Greece

**Keywords:** novel feed ingredients, methane emission, bioactive compounds, milk quality, meat quality, climate change, sustainable livestock

## Abstract

**Simple Summary:**

This review highlights the benefits of using valuable alternative feeds such as crop residues, silage, grasses, hay, browse, plant leaves, shrubs, and agro-industrial by-products in small ruminants’ diets. Alternative feeds can significantly improve the productivity and reduce carbon footprints and GHG fluxes of small ruminant farms, making them both environmentally friendly and cost-effective. Additionally, these alternative feeds possess antioxidant, antimicrobial, and antiseptic properties that can enhance the quality of the meat and milk produced.

**Abstract:**

Small ruminants, such as sheep (*Ovisaries*) and goats (*Capra hircus*), contribute to approximately 475 million metric tons of carbon dioxide equivalent (MtCO_2_e) greenhouse gas (GHG) emissions, accounting for approximately 6.5% of the global emissions in the agriculture sector. Crop residues, silage, grasses, hay, browse, plant leaves, shrubs, agro-industrial by-products, poultry litter, and other alternative feed sources are frequently utilized for small ruminant production. The use of these valuable alternative feeds can significantly improve animal productivity and reduce carbon footprints and GHG fluxes, making it both environmentally friendly and cost-effective. Additionally, these alternative feeds possess antioxidant, antimicrobial, and antiseptic properties that can enhance the quality of the meat and milk produced. By impacting the bacteria involved in ruminal biohydrogenation, alternative feeds can reduce methane emissions and contribute to a decrease in the carbon footprint. Overall, the use of alternative feed sources for small ruminants generally improves their apparent nutrient digestibility and productivity, and has an impact on the production of greenhouse gases, especially methane. Finally, this review recommends evaluating the economic analysis of reducing methane emissions in small ruminants by utilizing different feed sources and feeding techniques.

## 1. Introduction

Increased atmospheric emissions of greenhouse gases (GHGs), such as nitrous oxide (N_2_O), carbon dioxide (CO_2_), and methane (CH_4_), are the primary cause of climate change. These emissions result in unpredictable and erratic rainfall, floods, and droughts [1]. In 2020, agriculture contributed 5865.47 MtCO_2_e, representing 12.34% of its total emissions (47,513.15 MtCO_2_e), including land-use change and forestry [2,3]. In terms of regional distribution, Africa, South America, and Asia dictate the agricultural emissions, while Europe, North America and Oceania have the lowest emissions. As far as specific countries are concerned, the top 5 places on the list of the highest emissions from agriculture are occupied by India, China, Brazil, the USA, and Indonesia [3]. 

More than 83% of the total agricultural emissions are due to livestock emissions. Enteric fermentation is considered the biggest contributor (about 5.5 MtCO_2_e) to livestock emissions, followed by manure left in pasture (4.5 MtCO_2_e) [2]. Small ruminants, such as sheep (*Ovis aries*) and goats (*Capra hircus*), contribute approximately 475 MtCO_2_e to greenhouse gas emissions, constituting approximately 6.5% of the global emissions from the agriculture sector. The combined production of meat and milk from sheep and goats amounts to around 254 and 175 MtCO_2_e, respectively [4].

In the same way, several studies have predicted a further future decrease in total annual rainfall by 15–30% [5] and the expansion of desert climates. This expansion is attributed to increasing temperatures and decreasing precipitation. Moreover, these effects are likely to be “severe, pervasive, and irreversible” in the years to come [1,6,7,8,9]. Such changes can negatively affect livestock production and crop yields and threaten food and nutrition security [3].

In order to deal with these effects, there is an urgent need to transform agriculture, livestock farming, and food systems towards more sustainable production methods that respect the environment and meet consumers’ expectations while providing substantial income and good working conditions to the local farmers [10,11,12,13,14]. The reduction in carbon footprints and greenhouse gas fluxes, the production of environmentally friendly and healthier food products, as well as the genetic conservation and preservation of local breeds that are well-adapted to the local environment, are potential strategies that can be profitable and can safeguard natural resources for future generations [8,15,16,17,18].

To achieve these goals, among the selected strategies, research on livestock and animal production focuses on alternatives to feed, utilizing valuable wild plant genetic resources and agro-industrial by-products as potential feed alternatives, either as replacements or supplements. The development of enriched feed using tomato pomace (peels and seeds), faba beans, and pea by-products in the food industry, characterized by high protein levels, vitamins, and minerals, can serve as a potential auxiliary to pasture or as a nutrient supplement for mixed feed [19,20,21]. Shrubs, agro-industrial by-products, and aromatic plants have been explored as potential feed alternatives to enhance animal performance [22,23,24] and improve the quality of their products to reach consumer demand for safe and high-quality foods [24,25,26]. These effects are attributed to their richness in numerous bioactive compounds, such as phenolic compounds, providing antioxidant activity to reduce meat and dairy products’ oxidation and extend their shelf life [27,28]. Almond by-products could be potential alternatives to some forages given their richness in fiber; extracts prepared from whole almond seed, brown skin, shell, and green shell cover (hull) possess potent free-radical-scavenging capacities [29]. Additionally, the incorporation of sesame meal (46% crude protein (CP)) in ewes’ feeding (at 10 and 20%) enhanced the digestibility of both CP and fiber, consequently improving the average daily gain, feed conversion ratio, and cost of feed/kg gain in growing lambs when compared with diets that did not contain sesame meal [30]. Other plant-origin sources can be used as alternatives to dairy feed, such as pigeon pea (*Cajanuscajan* L. Millsp) leaves [31], *Sesbania sesban* leaves [32], olive cake [33], and apricot almond cake-meal [34]. These alternatives can improve the fat profile with less saturated and more polyunsaturated and monounsaturated fatty acids (e.g., 18:1 *trans*-11 fatty acid) [33] without affecting the milk yield or composition [31].

The overall objective of this review was to assess the effects of alternative feed resources utilizing valuable wild plant genetic resources and agro-industrial by-products on small ruminants’ productivity, product (milk and meat) quality, methane emissions, rumen digestibility, and farms’ economic performances.

## 2. Alternative Feed Resources in Small Ruminant’s Nutrition

Conventional feed resources have historically been used to meet the nutritional needs of small ruminants, such as grains, legumes, and forages. However, their wide use and increasing demand raise issues with respect to sustainable land and water use, greenhouse gas emissions, and the intensifying competition for arable land between food, feed, and fuel [35]. Global horticultural waste, in several cases, also results in landfills, increasing economic losses and environmental pollution. According to Ridolfi et al. [36], approximately one-third (1.3 billion tons) of food produced globally is wasted each year, while food waste was responsible for approximately 55 million metric tons of CO_2_ equivalent emissions from municipal solid waste landfills in the United States in 2020 [37]. Therefore, there is a need for more sustainable feed production, and the utilization of novel resources is crucial. There are currently various types of alternative feed resources for small ruminant nutrition and greenhouse gas emission reduction [38,39,40]: (1) agro-industrial byproducts/co-products, such as milling, sugar industry byproduct/molasses, starch alcohol, or plant oil, which stand out for their nutritional, economic, and environmental benefits; (2) forage legumes; (3) insect feeding; and (4) horticulture food waste.

In terms of agro-industrial byproducts from the brewing industry, brewer’s grain is considered a good source of protein since it has a crude protein content (CP) ranging from 19–31% on a dry matter basis (DM). Moreover, brewer’s yeast is also high in protein (36–50% on a DM basis), has a valuable amino acid profile similar to soy [41], and has been shown to be effective in replacing maize meal in sheep [42]. On the other hand, dried distillers’ grains (DDGs) are primarily made from maize, but they can also be produced from wheat, barley, rye, or a combination of these grains. They are high in protein, fiber, fat, and soluble sugars, and they are also rich in phosphorus, zinc, and potassium. Moreover, their fiber content helps to reduce ruminal acidosis in high-grain diets [43].

There are also many byproducts from the milling industry that could be used in small ruminants’ diets. Maize and wheat gluten meals are two high-protein, medium-energy feed materials that, according to some researchers, could contain up to 60–75% [44] or 75–80% CP [45], respectively. Kernel cake has also been used as a source of protein and energy, and has produced promising results in a high-concentrate feedlot for goats [46]. Other sources of nutrients that have been used include palm fiber, chickpea flour, rape seed meal, and cotton seed meal, which could be better described as energy feeds. 

Legume forages are known energy and protein sources. They include species like Leucaena, Sesbania, pigeon pea, lucerne, and clover, and they could be used as cut fodder, grazed pasture, or even as silage and fermented feeds as alternatives to conventional dry hay. They have been shown to increase milk production, improve reproductive performance, fix nitrogen, and enhance soil fertility [47]. 

Regarding insect feeding, studies on incorporating insects into small ruminant diets have shown promising results in terms of protein content and digestibility. Insect farming is also considered environmentally friendly and helps to reduce reliance on traditional protein sources such as soy. Insect protein’s lower ruminal N degradation may improve N utilization efficiency, and, thus, productivity, while decreasing N excretion into the environment [48]. Insect meal provides a highly digestible protein source, essential amino acids, and beneficial fatty acids, contributing to better animal performance and weight gain. It has a well-balanced nutritional profile that includes important amino acids, vitamins, and minerals [49]. For example, black soldier fly (BSF) larvae could replace soybean meal in creep feed for post-weaning goat kids without affecting weight gain or blood profiles. However, because of the chitin content in the frass, using BSF frass in the fattening goat ration resulted in lower digestibility of DM and organic matter (OM). However, the lactic acid bacteria (LAB) found in black soldier fly larvae grown on chicken manure have the potential to serve as probiotics for ruminants [50].

Making use of horticultural waste is an excellent way to reduce food–feed competition and landfill waste. Fruit and vegetable waste could be a promising alternative feed biomass for sustainable and clean animal production, opening a new avenue for safe utilization and promoting a greener environment. The amount of total soluble sugars in fruit waste is 27% higher than in vegetable waste. Total antioxidant capacity, flavonoids, and total phenols are 26.2, 103, and 71.8% higher in fruit waste than in vegetable waste, respectively. Both fruit and vegetable waste are rich in macro- and micro-nutrients; however, fruit waste contains more K, Fe, and Cu, whereas vegetable waste is high in Ca, K, S, and Na [51].

### 2.1. Effects on Methane Emission

Methanogenesis has been playing a significant role in digestion, producing methane as a byproduct of metabolism in ruminant animals, and this process has been a significant contributor to greenhouse gas emissions [52]. Various mitigation strategies have been developed to reduce methane (CH_4_) emissions, including dietary modifications involving lipids, essential oils, and algae [53,54]. Rumen microorganisms, such as bacteria, protozoa, and fungal zoospores, are linked to rumen fermentation efficiency. Methane losses appear relatively constant (6 to 7% of gross energy (GE) intake) for diets containing 30 to 40% concentrate, and then rapidly decrease to low values (2 to 3% of GE intake) for diets containing 80 to 90% concentrate [55]. Concentrates high in starch (wheat, barley, and maize) have a greater negative impact on CH_4_ production than fibrous concentrates [56]. Lima et al. [57] reported that when the level of concentrate inclusion in the diet exceeded approximately 50%, goats’ absolute CH_4_ emission decreased. Feeding orange leaves and rice straw to Murciano–Granadina goats reduced CH_4_ emissions without affecting energy balance [58].

While feeding cellulosic material increases enteric CH_4_ emissions, the amount depends on the forage source, chemical composition, and digestibility. This variation allows for CH_4_ reduction through dietary management. As a result, it is critical to consider the implications of the various options presented above when discussing CH_4_ mitigation. Local feed resources have been used as alternative feed additives for the manipulation of rumen ecology, with promising results for replacement in ruminant feeding, in addition to feed formulation and feeding management. The effects of feed additives are presented as follows. Saponins are abundant in Camellia Sinesis, Yucca schidigera, Quillaia Saphonia, and Meidcago sativa. Saponins, which are natural detergents found in many plants, have an indirect effect on methane emission output by slowing methanogenesis, increasing the expression of methanogenesis-related genes, and decreasing methanogen abundance [59]. Commercially available extracts of condensed tannins and saponins may exist. Tannin-rich plants and extracts have been shown in studies to reduce methane production [60]. van Gastelen et al. [61] reported a 34% increase in dry matter intake of tannin-rich forage in sheep, and the CH_4_ yield (g/kg DMI and % GEI) decreased by 23 and 36%, respectively. Substituting tannin-rich forages for grass pastures or grass silage reduces CH_4_ emissions from both dairy cattle and sheep. It is worth noting that the response to tannin feeding varies greatly depending on the tannin source, type, and molecular weight, as well as the methanogen community present in the rumen and its concentration in forage and feed supplements (20 g/kg of dry matter intake, (DMI)) [62].

Flavonoids used as feed additives include celery, parsley, red peppers, chamomile, mint, ginkgo biloba, and citrus fruit peels. Similarly to tannins, flavonoids are classified as secondary plant metabolites. They have the capability to reduce protozoa and methanogen populations, inhibiting methanogenesis. Flavonoids have a significant potential to reduce methane emissions, but further research in in vivo trials is needed. Dietary fat appears to be a promising nutritional option for suppressing ruminal methanogenesis while having no effect on other ruminal parameters [55].

Lipids used as feed additives represent a viable strategy for reducing CH_4_ emissions (a 14% reduction in long-term CH_4_ emissions was achieved when lipid sources were added to supply 34 g fat/kg of DM) [63]. However, factors such as the physiological stage of the animal, the composition of the basal diet’s lipids and other nutrients, and the fatty acid profile of the supplemental oil can influence the maximum oil inclusion in ruminant diets. High lipid concentrations (>6% DM) in feed can reduce feed and fiber digestibility, potentially increasing OM [52].

Plant materials such as flowers, seeds, buds, leaves, herbs, wood, fruits, twigs, garlic, eucalyptus, clove, rosemary, thyme, paprika, juniper, ginger, and roots are used to extract plant essential oils (EO), which are volatile, oily, and aromatic liquids. Some essential oils can influence rumen fermentations and reduce CH_4_ production in vitro. Encouragingly, certain studies have demonstrated remarkable reductions in CH_4_ emissions of up to 90% [64]. However, with the increased use of EOs, there is a need for regulations that include maximum permissible limits, toxicity considerations, and the capacity of EOs against specific methanogens, ensuring that they do not adversely affect other groups of microorganisms in the rumen.

Furthermore, algae have become a subject of research aimed at reducing ruminant CH_4_ emissions, with positive findings in methane emission mitigation as a CH_4_ mitigation additive. Macroalgae supplementation has emerged as a promising tool for reducing ruminant enteric methane emissions [65]. Furthermore, this additive increased the economic efficiency of treated animals by approximately 53.13% [66]. However, it is important to note that long-term oral exposure of animals to high concentrations of Bromoform (CHBr_3_) can result in liver and intestinal tumors. On a different note, the inclusion of up to 10% fruit and vegetable waste in sheep diets improved nutrient utilization and antioxidant status while lowering GHG emissions. These wastes can also serve as a valuable preformed water source for livestock in dry areas, resulting in a net reduction in potable water consumption of 21.78 and in fruit and vegetable waste of 13.92% [51].

### 2.2. Effects on Rumen Digestibility

Several academics have investigated the use of substitute feed sources for small ruminants to improve apparent (in vivo) nutrient digestibility and mitigate greenhouse gas production, particularly methane (CH_4_) emissions. Consequently, Table 1 presents the apparent nutrient digestibility of alternative feed resources used to feed small ruminants.

#### 2.2.1. Grasses and Hay

Although Brhanu and Gebremariam [67] reported that an increase in the amount of *khat* leftover meal decreased the apparent digestibility coefficient of DM and nutrients in sheep, in most cases, the use of grasses and hay in small ruminant diets improves the rumen digestibility. For example, using cactus as a supplement for small ruminants on poor roughages like straw has been shown to increase the amount of straw consumed, to improve diet digestibility, and to boost microbial activity [68]. In another report, the inclusion of 60% tomato pomace in the diet of lactating goats significantly increased the apparent digestibility values, specifically 0.85% for ether extracts and non-fibrous carbohydrates [69]. Wadhwa et al. [70] conducted in vivo studies on goat bucks using a diet containing 0–50% bottle gourd pulp (*Lagenaria siceraria*) pomace in iso-nitrogenous and iso-caloric concentrate mixtures supplemented with green fodder (50:50%), and found that the fungal population in the rumen increased significantly, while the bacterial and total protozoal population decreased significantly as the amount of pulp in the diet increased. Although the N-retention in bucks remained unaffected, the digestibility of CP decreased, whereas that of acid detergent fiber (ADF) and cellulose rose dramatically. It was determined that adult ruminant concentrate mixtures can contain up to 50% bottle gourd waste (pulp). With complete diets containing poultry litter (35%) tested in Nellore and native rams, sheep exhibited higher DM spontaneous feed intake and DMI, digestible crude protein (DCP), and total digestible nutrient (TDN) intake per kg than goats [71].

Improved dry matter intake and significant total digestibility of DM, OM, and NDF were observed in adult sheep fed a total mixed ration (TMR) containing moist okara silage [72,73]. A mixture of alfalfa hay and concentrate (50:50%) fed to Barki sheep and Balady goats resulted in 58.5 and 53.4% DM digestibility, 58.4 and 53.5% OM digestibility, 68.1 and 62.6% CP digestibility, and 58.4 and 51.1% neutral detergent fiber digestibility in sheep and goats, respectively [74]. Otoni et al. [75] fed sheep a variety of grass and legume hays and reported in vivo DM digestibility of 47.6, 53.4, 29.3, and 53.2% for Jiggs hay (*Cynodon dactylon*), Tifton-85 (*Cynodon* spp.), stylo (*S. capitata × S. macrocephala*), and alfalfa (*Medicago sativa*) hays, respectively. Yang et al. [76] fed 5, 10, and 15% sorghum hull as an alternative feed source to growing goats and reported CP digestibility of 14.8%, 19.5%, and 16.8%. The authors indicated that sorghum hull was used as a feed alternative for growing goats due to its benefits for growth performance, nutrient digestibility, and plasma metabolites. Goats were fed four different types of grasses, including *Brachiaria-decumbens*, *Panicum maximum*, *Elephant grass*, and *Mini elephant grass*, each at 80% plus 20% rice bran, according to a study by Ismartoyo et al. [77]. In vivo neutral detergent fiber (NDF) and ADF digestibility were found to be 54 and 34.5%, 60 and 44%, 47.7 and 66%, and 27.5 and 48.9%, respectively. Finally, the authors came to the conclusion that feeding rice bran (20%) in a diet that included *Brachiaria-decumbens*, *Panicum maximum*, *Mini elephant*, and *Elephant grass* increased the feed’s digestibility and NDF/ADF intake values. Therefore, it could be concluded that the use of grasses and hays in small ruminant nutrition is highly promising and could improve the apparent digestibility of nutrients, thus reducing CH_4_ emissions.

#### 2.2.2. Fruit and Vegetable Waste

Small ruminants are adept at digesting lignocellulosic agro-industrial byproducts. Therefore, digestible food waste, such as fruit and vegetable byproducts, can also be utilized as a feed supplement for ruminants [78,79]. For example, when adult sheep were fed dietary combinations made from varying quantities of fruit waste, as conducted by Sahoo et al. [51], it was found that, without changing feed intake, the supplemented groups had considerably improved CP and DM digestibility by 7.3 and 7.6% and 5.5 and 7.2%, respectively. Carrot top hay, when replaced with 50% of berseem (*Trifolium alexandrium*) hay in the diet of Rahmani sheep, increased nutrient digestibility [80] because of its high nutritive value OM (977.6%), CP digestibility (62%), and TDN (73% on DM basis), in addition to an ME value of 10.2 MJ/kg DM. When Silivong et al. [81] added brewers’ grains (5% of the diet DM) to the diet of goats, they observed that the protein supplement, which was made up of cassava leaves and water spinach, respectively, increased the growth rate by 44 and 11% and the DM feed conversion by 25 and 5%. According to the authors, adding brewers’ grains, a fermented byproduct of making “beer”, to a diet that includes potentially harmful ingredients, like the cyanogenic glucosides found in cassava leaves, can have prebiotic effects. According to Arun et al. [82], the addition of 50% leftover jackfruit to the finger millet straw silage diet of Mandya lambs enhanced the nutrient content by adding nitrogen and fermenting the mixture with *Saccharomyces boulardii* and *Lactobacillus acidophilus*. However, the study found that this did not alter the nutrients’ digestibility or the amount of DM the lambs consumed. Similarly, in Nkosi et al. [83]’s study, South African Dorper lambs were fed ad libitum with discarded cabbage (*Brassica oleracea* var *capitata*) at concentrations of 0, 100, 150, and 200 g/kg. The higher the cabbage concentration in the diet, the lower the DM intake and lamb growth performance were. The addition of cabbage to the lambs’ diet had a negative effect on their feed conversion rate. Diets containing cabbage caused the lambs to digest less DM, OM, and NDF than diets without it. Therefore, the general use of fruit and vegetable byproducts could have a beneficial effect on rumen digestibility, but since this is not the case for every byproduct, each one should be tested and evaluated before use.

#### 2.2.3. Silage

While silages are widely used in ruminant nutrition, in a study conducted by Gholami-Yangije et al. [84], when sunflower (*Helianthus annuus*) residue silage was substituted for alfalfa hay and maize silage in the diet of Mohabadi dairy goats, the researchers observed a decrease in the digestibility of both DM and OM as the amount of sunflower residue silage (SRS) in the diet increased. It was suggested that the lower DM and OM digestibility at higher SRS inclusion levels could be a secondary effect of lower CP digestibility. On the other hand, when whole corn plant silage was added to the Napier grass diet for goats at inclusion levels of 25% and 50%, Khaing et al. [85] observed a linear increase in nutrient digestibility. This increase may be attributed to the fact that whole corn plant silage has fewer structural carbohydrates than Napier grass, making it more susceptible to rumen microbial degradation. When 25% or 50% of the whole corn plant silage was substituted for the Napier grass diet, the apparent digestibility of CP also increased linearly. This may have been brought on by the diet’s high nitrogen absorption. The whole corn plant silage incorporated in Napier grass showed a significant decline at the 75% inclusion level, which may have been caused by the combined diet’s unfavorable associative effect. Ultimately, the study concluded that feeding a combination of corn silage with Napier grass at 50% and 100% resulted in high nutrient utilization. According to Munguía-Ameca, et al. [86], it is possible to incorporate up to 20% of ensiled coffee pulp in Pelibuey lambs’ diets without affecting their productivity, ruminal fermentation, nutrient digestibility, or carcass and meat characteristics. The study also demonstrated that adding up to 20% ensiled coffee pulp to the diets of fattening lambs influenced antioxidant compounds in the diets, antioxidant capacity in blood serum, and the digestibility of CP (74.99% in vivo digestibility). When sheep were fed cassava leaf silage (CLS) in four different combinations by Sudarman et al. [87], the consumption of CP, fat, crude fiber, and total digestible nutrients was significantly impacted, but the consumption of DM was not significantly affected. A 20% concentration or CLS level was found to improve feed efficiency and body weight. The total number of bacteria did not differ significantly; however, the total number of protozoa rose with the concentration. In summary, the performance of sheep fed 20% cassava leaf silage was significantly enhanced, approaching the level attained by feeding concentrate. Abo-Donia et al. [88] fed ensiled rice straw by water (RSW), molasses plus urea (RMU), whey (RWh), and untreated rice straw (URS) to sheep as part of their study. The highest digestibility values for DM, OM, CP, EE, NDF, and ADF were found in the ensiled RWh and RMU, and these values were significantly higher than those found in RSW. Their study’s findings suggest that rice straw ensiling could be beneficial if milk whey or sugarcane molasses plus urea were added to increase the nutritional value and enhance palatability, feed consumption, and digestibility. Even though ensiling rice straw with additional whey performed better than ensiling it with additional molasses and urea, the findings suggest that any of these additives can be utilized to enhance the utilization of rice straw.

#### 2.2.4. Browse and Leaves of Plants

Grazing is a standard practice in most production systems for small ruminants due to the fact that these animals can utilize a wide range of wild plants for feeding. Haruna et al. [89] investigated the CP digestibility of four semi-arid browse plants in sheep, observing an average of 87.55%. They reported varying CP digestibility for different browse plants: *Ficus sycomorus* (94.18%), *Ziziphus Mauritania* (92.41%), *Balanites aegyptica* (92.37%), and *Celtis integrifolia* (71.25%). They showed that these peruse plants act as an elective feed source particularly during the long dry period in the semi-parched zone due to their high potential protein digestibility. In a study conducted by Avornyo et al. [90], six fodder species (*Annona senegalensis, Ficus gnaphalocarpa, Pericopsis laxiflora, Pterocarpuserinaceus, Afzelia africana,* and *Arachis hypogaea*) were fed to dwarf goats in West Africa. The study found that the digestibility indices of four of the feeds had DM contents of 60 to 75%, and the digestibility indices of browse species, like groundnut haulm, were within the usual range for recommended fodder species. Increased nutrient digestion of DM, OM, protein digestibility, and lipid digestibility was observed in goats supplemented with 6% neem leaf and 15% polyethylene glycol in the concentrate [91]. Mekuriaw and Asmare [92] examined the digestibility of concentrate mixtures up to 75% substituted with leaves from the fodder tree, *Ficus thonningii* (FTL), in the diets of Washera lambs. They found that these treatment diets had better digestibility coefficients, with the exception of the 100% FTL supplement group. Ultimately, it was determined that the leaves of the native fodder tree FTL could be utilized as a substitute concentrate mixture up to 75% in order to enhance performance when fed to Washera sheep in their natural pasture hay diet. Adelusi, et al. [93] fed ground tree leaves (*Azadirachta indica*, *Newbouldialaevis*, and *Spondias mombin*) to dwarf West African goats and discovered that the addition had no effect on the digestibility of DM, OM, or fiber fractions. Nonetheless, there was a notable decline in the animals’ ability to digest ash and CP when fed ground leaves. *A. indica* and *S. mombin,* when added to the animals’ diets, caused the goats’ nitrogen balance to decrease. It can be concluded that, while adding *N. laevis* to goats’ diets improved their CP digestibility, nitrogen balance, and retention, feeding them ground leaves of *S. mombin* greatly increased their weight gain. Ajagbe et al. [94] carried out a feeding trial to assess the nutrient digestibility and nitrogen balance of growing African Dwarf goats fed supplemented cassava peel meal diets. Based on the nutrient digestibility and nitrogen retention characteristics of cassava peels, the study concluded that supplementing nitrogen to the diets of goats may enhance the productive performance of small ruminants.

#### 2.2.5. Agro-Industrial By-Products and Other Alternative Feed Sources

Ahmed et al. [95] evaluated the effects of substituting up to 40% of noug seed cake with poultry litter for Arsi Bale goats, but they did not find a significant difference in the in vivo digestibility of DM, OM, CP, neutral detergent fiber (NDF), and acid detergent fiber (ADF). Lastly, they suggested that this feed compounding, which incorporates up to 40% poultry litter, is economical and improves the growth performance of goats. Due to increased nutritional digestibility, linseed oil supplementation at 4% in a fattening diet increased the average daily gain (ADG) by roughly 29% and decreased the feed conversion ratio (FCR) by roughly 18% in both goats and sheep [96]. Hao et al. [97] substituted flax seed meal (FSM) for soybean meal (SBM) in the diets of sheep at four different levels, and the findings showed that FSM can successfully replace SBM in the diets of fattening sheep, with 12.0% being the ideal ratio in this scenario. The study demonstrated that adding FSM to sheep diets in place of certain SBM can improve the apparent digestibility of DM and NDF throughout the entire digestive tract. Mengistu et al. [98] studied the effects of feeding Bonga sheep Rhodes grass hay on feed intake, body weight change, and the digestibility of noug seed (*Guizotia Abyssinica*) cake substituted with dried mulberry and *Vernonia* mixed leaf meal. They showed that a meal consisting of mixed dried mulberry and *Vernonia* leaves can replace NSC as a protein supplement by up to 75%, providing yearling Bonga sheep with the best possible DM and nutrient intakes, as well as body weight gain. This study also emphasizes the benefits of feeding ruminants meals consisting of mixed leaves from *Vernonia* and dried mulberries as a supplement to their basal diet of fibrous feeds. In a published study, Hao et al. [99] examined the effects on growth performance, rumen microbial protein synthesis, and nutrient digestion of fattening lambs fed a combination of soybean hulls and corn bran in place of some corn and corn stover. Three groups of randomly selected thin-tailed crossbred ram lambs were formed: 0% corn bran and 9% soybean hulls; 17% corn bran; and 17% soybean hulls. The findings showed that soybean hulls and corn bran together can successfully replace some of the corn and corn stover in finishing lambs’ diets, improving nutrient absorption and growth efficiency.

Overall, feed digestibility is an important indicator that can be used as a guide to determine the amount of nutrients and feed that can be absorbed in the gastrointestinal tract [100]. In extensive ruminant production systems, as opposed to intensive ones, there is significantly greater potential for ruminant performance gains and methane emission mitigation through appropriate forage supplementation and feed selection to enhance forage and total diet digestibility [101,102].

**Table 1:** Digestibility of alternative feed resources for small ruminants.

Alternative Feed Resources	Apparent Digestibility (%) *					
	DM	OM	CP	EE	NDF	ADF
*Azadirachta indica* ^1^	69.6	71.0	76.2	65.3	70.8	44.7
*Spondias mombin* ^1^	71.3	72.1	71.7	73.0	72.3	49.3
*Newbouldia laevis* ^1^	71.8	72.0	81.3	78.9	72.8	59.9
*Ziziphus mauritania* ^2^	98.4		92.4	58.3		
*Balanites aegyptica* ^2^	65.2		92.4	69.3		
*Ficus sycomorus* ^2^	58.1		94.2	64.9		
*Celtis integrifolia* ^2^	64.2		71.3	70.8		
*Afzelia africana* ^3^	53.3	52.0	70.8		46.7	28.1
*Ficus gnaphalocarpa* ^3^	69.1	63.7	76.3		70.4	64.2
*Annona senegalensis* ^3^	69.5	71.6	61.3		62.3	67.1
*Arachis hypogaea* ^3^	73.9	75.5	75.2		67.5	68.1
*Pericopsis laxiflora* ^3^	62.8	60.7	60.5		60.1	64.3
*Pterocarpus erinaceus* ^3^	41.8	54.4	71.9		60.6	53.5
Cassava peels ^4^	95.3	90.0	93.0	90.0		
Sorghum hull ^5^	57.3	68.0	54.2		57.7	42.3
Poultry litter ^6^	82.6	77.1	7.37		38.1	27.7
BottleGourd (*Lagenaria siceraria*) Pomace ^7^	54.2	57.3	57.7		46.6	40.4
Corn silage ^8^	71.0	72.8	63.7		61.3	53.1
Fruit waste ^9^	55.5	57.8	62.5	78.0	49.4	42.5
Vegetable waste ^9^	56.4	58.6	62.7	78.2	51.0	41.7
Cabbage ^10^	64.0	65.0		53.0	47.0	
*khat* leftover meal ^11^	50.9	64.3	82.0		44.9	77.4
Soybean meal ^12^	57.0	61.3	67.4		41.7	31.6
Flax seed meal ^12^	57.6	61.7	63.3		42.0	33.1
Noug seed cake ^13^	68.8	71.5	78.6		63.8	48.0
Mulberry and Vernonia mixed leaves’ meal ^13^	64.4	66.8	73.9		60.1	41.5

* DM: dry matter, OM: organic matter, CP: crude protein, EE: ether extract, NDF: neutral detergent fiber, ADF:acid detergent fiber. Adapted from: ^1^ Adelusi et al. [93], ^2^ Haruna et al. [89], ^3^ Avornyo et al. [90], ^4^ Ajagbe et al. [94], ^5^ Yang et al. [76], ^6^ Ahmed et al. [95], ^7^ Wadhwa et al. [70], ^8^ Khaing et al. [85], ^9^ Sahoo et al. [51], ^10^ Nkosi et al. [83], ^11^ Gebremariam and Brhanu [67], ^12^ Hao et al. [97], ^13^ Mengistu et al. [98].

## 3. Alternative Feed Resources and Small Ruminants’ Product Quality

### 3.1. Milk Production

For several decades, studies have been conducted to assess the effects of alternative feeds on milk yield and quality in small ruminants, as well as on other animal performance parameters [103,104]. Given their chemical composition, which is rich in bioactive molecules and secondary metabolites such as phenols and tannins, shrubs, legume seeds and pods, and local agro-industrial by-products, such as olive pomace, tomato spent grains, and date scraps, which are inexpensive and widely available, these feeds can influence milk production (Table 2).

#### 3.1.1. Wild Plants, Leaves, and Shrubs

It is eminent in the literature that tanniniferous resources are the main suppliers of condensed tannins. They are widely distributed in the plant kingdom and present in angiosperms and gymnosperms. Plants rich in condensed tannins are abundantly found within the legume family, particularly in numerous forage species such as sainfoin (*Onobrychis viciifolia*), sulla (*Hedysarium coronarium*), pedunculate, and horned trefoil (*Lotus pedonculatus* and *Lotus corniculatus*), as well as several Acacia species [105]. The effect of tannin-producing feeds on milk yield and the composition of milk fat and protein varies considerably depending on the concentration of tannins present in the feed, and results frequently display inconsistency. Condensed tannins in high concentrations generally exert negative effects on animal performance. However, moderate concentrations can have positive effects [106]. In this context, there have been reports indicating that tannins can enhance the fatty acid (FA) profile of milk by influencing ruminal FA metabolism. This, in turn, leads to an increase in the outflow of beneficial fatty acids (such as CLA and n-3 PUFAs) from the rumen, ultimately improving their concentrations in milk [107].

Wang et al. [108] examined the effect of tannin concentration on the yield and composition of sheep milk. Ewes grazing *Lotus corniculatus* with moderate tannin amounts, with or without polyethylene glycol (PEG), were studied. PEG is a binding agent capable of forming complexes with condensed tannins without disrupting animal digestion [109]. The supplementation had no impact on milk yield until the fifth week of lactation. However, from the sixth to the eleventh week, the milk yield increased significantly. At week 11, the milk yield was significantly higher in ewes fed the PEG-free diet. The milk fat content was higher in the PEG-supplemented group, showing the lowest milk yield. Sulla flexuosa (*Hedysarum flexuosum* L.), a native leguminous plant in Mediterranean regions, was employed as an alternative protein source in goat diets, replacing alfalfa, with the primary aim of enhancing forest rangeland nutrition [110]. The observed rise in polyunsaturated fatty acid (PUFA) levels in the milk of animals fed sulla could be attributed to the presence of tannins. These tannins may have influenced the activity of rumen microflora, consequently reducing the biohydrogenation process of PUFA [106].

In a more recent study, Jerónimo et al. [111] reported that incorporating extracts abundant in condensed tannins from rockrose (*Cistus ladanifer* L.) and quebracho (*Schinopsis lorentzii*) into the diets of Serpentina goats resulted in a reduction in branched-chain fatty acids and C18:1 *trans*-10 in milk fat. Notably, dietary supplementation with condensed tannins did not impact the intake of polyunsaturated fatty acids (PUFA), 18:1 *trans*-11, 18:2 *cis*-9, or *trans*-11, nor did it impact the milk yield. In Canindé, Repartida, and Saanen goats, conventional feed enriched with condensed tannins from *Acacia mearnsii* (50 g/kg DM) has been shown to increase the levels of C14:1, *cis*-9, C18:2n6, C18:3n6, C18:3n3, polyunsaturated fatty acids (PUFA), and long-chain fatty acids. Simultaneously, it reduces the levels of C12, C14, and ω6/ω3, as well as the atherogenicity index, in milk fat, all without any modifications to nutrient intake or animal performance [112].

#### 3.1.2. Legumes and Algae

As shown in the Table 2 replacing cereals with grain legumes as alternative protein resources in ruminant diets generally enhances the milk yield and improves the lipid profile [113,114]. In Chios ewes, the inclusion of Camelina sativa seeds at 16% (DM) resulted in a significant reduction in milk fat and an improvement in milk quality from a human health perspective. This improvement was achieved by altering the content of saturated fatty acids, adjusting the proportions of α-linolenic acid (C18:3 n-3), C18:2 cis-9, and trans-11 (CLA), and modifying the ω6/ω3 ratio. Additionally, there was a reinforcement of milk oxidative stability, evidenced by a significant increase in the activity of catalase (CAT), superoxide dismutase (SOD), malondialdehyde (MDA), protein carbonyls, and glutathione peroxidase (GSH-Px) [115]. In a more recent study, the Spirulina, an edible blue-green algae rich in bioactive compounds such as vitamins, minerals, antioxidants, and γ-linolenic acid, was used to enrich the feed of Chios ewes at a rate of 15 g/ewe/day. This dietary intervention demonstrated an improvement in the potential oxidative status of both the ewes’ organisms and their milk, without exerting any effects on milk yield or chemical composition [116].

#### 3.1.3. Agro-Industrial By-Products

The incorporation of agro-industrial by-products into ruminant diets can lead to reduced feeding costs and environmental impacts, contributing to improvements in animal performance and product quality [103]. Utilizing unmarketable tomato (*Solanum lycopersicum* L.) or olive by-products supplemented with sunflower oil (20 g/kg DM) as alternative feeds has been found to modulate the lipid profile in Murciano–Granadina dairy goats. This approach results in an increase in C18:1 *trans*-11, C20:2, and conjugated linoleic acid, and a decrease in C4:0, C6:0, and C8:0. Furthermore, it leads to an overall increase in total saturated fatty acids and a reduction in C18:1 n-9 *cis*, as well as both total mono- and polyunsaturated fatty acids [117]. Moreover, incorporating olive by-products up to 20% (DM) in the diets of Damascus dairy goats has been associated with heightened expression of genes involved in lipogenic pathways (including de novo synthesis, fatty acid uptake and transport, and fatty acid desaturation), along with their regulatory elements such as transcription factors. Specifically, there was increased expression of *SLC2A1*, *VLDLR*, and *FABP3* in the mammary glands. Additionally, there was an upregulation of *FASN* and *SLC2A1* genes in adipose tissue [118]. At the same concentration of 20% (DM), olive by-products exhibited a linear increase in the concentration of unsaturated fatty acids (FA) of up to 20%, monounsaturated FA up to 23%, polyunsaturated FA up to 11%, and rumenic acid (CLA *cis*-9, *trans*-11) up to 61% in the milk of Chios ewes. Consequently, this led to a reduction in the atherogenicity and thrombogenicity milk indices by 31% and 27%, respectively [119].

**Table 2:** The effect of some alternative feeds on the quality and quantity of milk from small ruminants.

Alternative Feed	Main Findings	Reference
Sulla flexuosa (*Hedysarum flexuosum* L.)	-Antioxidant capacity (+) -Unsaturated fatty acids (+)	Boukrouh et al. [110]
Linseed	-Reduction in milk urea -n-3 FA and CLA (+)	Maamouri et al. [120]
Leaves of *Acacia cyanophylla*	-Reduction in milk urea	Maamouri et al. [120]
*Camelina Seed*	-Antioxidant capacity (+) -Unsaturated fatty acids (+)	Mierlita and Vicas [121]
Olive by-products silage Tomato surplus silage	-Saturated fatty acids (−) -Unsaturated fatty acids (+)	Arco-Pérez et al. [117]
Tomato fruits, citrus pulp, brewer’s grain, and brewer’s yeast	-Casein content (+) -Unsaturated fatty acids (+) -Saturated fatty acids (−)	Romero-Huelva et al. [122]
*Cynodondactylon* L.(Tifton) hay	-Milk production (+) -Fat content (+)	Mizael et al. [69]
Date waste	-Milk yield (+)	Boudechiche et al. [123]
Olive cake and tomato pomace	-Fat content (+)	Abbeddou et al. [33]

+ = improved/increased, − = declined/decreased.

### 3.2. Meat Production

The use of unconventional feeds, such as shrubs and plant by-products, as potential alternatives (either as a replacement or as a supplement) for enhancing animal performance and product quality has been explored [124,125]. In fact, when speaking about unconventional feeds, a special reference must be made to aromatic plants (APs), since their use in small ruminant feeding has shown numerous advantages in terms of meat quality and production. Studies have shown that the rearing of lambs on pastures rich in APs has been associated with an increase in slaughter weight and a decrease in carcass fat proportion [126,127], while when cull ewes were fed on AP-rich pastures, the fatty acid profiles were improved, the n-3 PUFA content was increased, and the lipid oxidative stability and meat color were improved [128]. On the other hand, when APs were incorporated into the diet, they tended to improve the tissular composition of carcasses and meat quality of young lambs [129]. 

Important quantities of aromatic and medicinal plant by-products are generated from the distillation industry, which could be valuable natural sources of antioxidants due to their richness in phenolic compounds [130]. Distilled leaves are discarded after the extraction of essential oils, whereas they could constitute an interesting alternative feed for small ruminants given their protein and energy content and their richness in bioactive compounds [123]. Rosemary distillation by-products were used in a lamb fattening trial, in combination with other local resources, as a substitute for commercial concentrate [131,132]. The results showed that the growth performances and meat physicochemical characteristics (pH, water cooking loss, color parameters, and protein content) were not affected by the diet, but the intake of these by-products showed their effectiveness in protecting meat against discoloration (high red index and chrominance during the storage period) and lipid oxidation (1.3 mg MDA/kg of meat). The inclusion of rosemary distillation by-products also increased the total polyunsaturated fatty acids and the ratio of polyunsaturated fatty acids to saturated ones.

Furthermore, the partial substitution of roughage by rosemary distillation by-products in cull ewes showed a decrease in branched-chain fatty acid content without changes in the sum of n-6 or the n-6/n-3 ratio. In addition, the meat from the rosemary distillation by-product diet retained its red color throughout the storage period. Concerning lipid oxidation, the lowest values were recorded in the rosemary by-product groups, remaining below 1.7 mg MDA/kg until the end of the storage period. Consequently, the level of oxidation of this meat was below the threshold (2 mg MDA/kg) for the detection of rancidity. The intake of rosemary by-products did not affect the meat’s sensory quality [133]. Similarly, the total substitution of hay by rosemary distillation by-products improved lambs’ growth performance, but did not modify carcass characteristics [124]; however, it retarded meat lipid oxidation during storage and improved meat color stability [24]. Hence, the meat α-tocopherol concentration from lambs receiving rosemary distillation by-products was four times higher (>6 μg/g) compared with the control ones (1.5 μg/g).

In addition to rosemary, the substitution of roughage or concentrate with myrtle distillation by-products in cull ewes showed that these residues could replace up to 87% of hay and up to 30% of concentrate in the ewes’ diet without showing any negative effects on body weight, carcass characteristics [130], or meat quality [26]. The chemical composition, pH, and color of the meat were not affected by the addition of myrtle residues. However, feeding the ewes these by-products resulted in meat richer in polyphenols and α-tocopherol (9 vs. 3 μg/g DM for myrtle and control groups, respectively). In addition, from the third day of meat storage, lipid oxidation was improved by the addition of myrtle residues, being lower for the two myrtle groups compared with the control one (0.51 vs. 1.11 mg MDA/kg meat).

On the other hand, the extraction of olive oil generates important quantities of by-products like pulp, skin, and water, which constitute environmental pollutants owing to their elevated organic composition. The valorization of these by-products in the form of animal feeds could reduce the expenses associated with waste management and animal feeding. In this context, the dietary incorporation of olive cake (280 g/day) into Barbarine lambs reared indoors or on a *Medicago arborea* rangeland did not alter the slaughter parameters, carcass traits, tissular composition, or meat quality, except for juiciness, which increased [134]. Similar findings were noted by Kotsampasi et al. [135] after the supplementation of partly destoned exhausted olive cake into the diet of Florina lambs’; no effect on growth performance, carcass weight, or intramuscular fatty acid profile was observed, whereas the carcasses’ qualitative traits were improved. Similarly, replacing 44% of the diet with a blend of agro-industrial by-products containing 18% corn distillers dried grains with soluble, 18% dried citrus pulp, and 8% exhausted olive did not change the growth performance, carcass weights, or yield [38], but increased the meat’s shelf-life (6 days of refrigerated storage) and improved its fatty acid profile by decreasing saturated and increasing polyunsaturated FA content [136]. Furthermore, the dietary incorporation of stoned olive by-products in the form of cake (35%) enhanced the oxidative stability of Appenninica lamb meat and improved the fatty acid composition of meat when combined with linseed (17% and 10%) [137]. This combination had no effects on feed intake, growth rates, or carcass characteristics; however, it increased the content of C18:3n-3 in intramuscular total lipids [138]. Additionally, the administration of polyphenols extracted from olive mill wastewater exhibited positive effects on Saanen goat kid meat. This administration resulted in enhancements in the fatty acid profile, specifically increasing oleic and conjugated linoleic acid contents and reducing malondialdehyde formation [139].

Transitioning to alternative agro-industrial residues, it is essential to emphasize that within the tomato juice and pulp industries, around 10% of the total tomato weight is designated as waste. The chemical composition of tomato pomace includes approximately 16% CP and 57% NDF [140]. Due to its favorable chemical composition and absence of acceptability issues, tomato pomace has emerged as a significant component in the diets of small ruminants [69,141]. Previous studies have explored the utilization of tomato residue in various forms, such as silage [142] or in combination with other residues [122]. Researchers have concluded that these dietary approaches do not adversely impact animal performance or product quality.

Moreover, the chemical composition of sesame seed meal varies according to the extraction method. The dry matter contents of sesame seed meal ranged from 83 to 96%, while the CP contents ranged from 23 to 46% [143]. Drawing a connection to this composition, the incorporation of sesame hulls in lamb diets (100 and 200 g/kg feed, respectively) improved the final weight and carcass characteristics, but led to similar meat oxidative stability and fatty acid profiles [144]. However, in another study, the effects of using sesame seed waste did not affect the growth performances nor the carcass characteristics of Karayaka lambs [145].

In a more recent study, de Castro et al. [146] found that incorporating oilseed cakes into the diets of lambs had significant effects on the lipid and protein composition of meat, specifically in the *Longissimus lumborum*. The inclusion of oilseed cakes resulted in decreased cooking losses and influenced various color parameters, such as L*, a*, b*, chroma, and hue angle. Additionally, it induced alterations in the total fatty acid (FA) composition and FA profile. Notably, there was a reduction in hypocholesterolemic fatty acids, although it did not have any discernible impact on indicators of atherogenicity, thrombogenicity, or cholesterolemia.

## 4. Alternative Feed Resources and Farms Economic Performances

Depending on the farm species, feed costs constitute a significant portion of livestock production expenses, ranging from 60% to 85% of the total annual cost inputs [45]. Therefore, the use of alternative feeding resources, which, in general, cost less than the conventional ones, could optimize efficiency and sustainability in livestock farming by reducing production costs [147]. However, in the relevant literature, it is rare to find data that provide a sufficient evaluation of the economic performance of alternative feeding strategies for the production of small ruminants. 

In Brazil, Silva et al. [148] reported that enriching sheep feed with wet brewery residue at 30% generated the highest profit (USD 0.27/kg of BW) and the lowest total expenses (USD 13,247.07). In a separate study, Lima-Cavalcanti et al. [149] evaluated the economic viability of including licuri oil in the diet of Santa Ines ewes at various levels. The recorded results indicated that the control diet yielded the highest gross revenue (USD 609.39); nevertheless, there was a loss (USD 50.96) in terms of economic performance indicators.

In northern Jordan, incorporating black cumin meal (*Nigella sativa*) as an alternative feed in Awassi ewes at 100 g/kg of dietary DM resulted in a decrease in feed costs from USD 399/ton to USD 353/ton, proving to be economically beneficial. Additionally, the utilization of this cost-effective feed positively influenced milk production. As a result, the cost of milk was significantly reduced with the alternative diet compared to the conventional one [104].

Similarly, Mahmoud and Bendary [150] reported a significant decrease in daily costs and a remarkable increase in economic efficiency by 57% through the utilization of a combination of black cumin meal (*Nigella sativa*) and sesame seed meal as protein sources in the rations of growing lambs. Moreover, the substitution of bovine cheese whey for traditional feed significantly reduced annual feedlot costs, decreasing expenses from USD 6790.04 to 4497.97 for finishing 240 lambs. This alternative feed not only ensured adequate performance in Morada Nova sheep, but also contributed to a reduction in labor costs and an improvement in the sale price of lambs [151]. Cassava root sieviate and cassava leaf were used as supplements to Panicum feed for West African Dwarf goats in Nigeria. The results demonstrated a decrease in feed costs alongside an increase in weight gain, contributing to enhanced farm profitability [152].

## 5. Concluding Remarks

Confronted with environmental constraints and food insecurity driven by demographic growth, there is an imperative need to cultivate more sustainable animal production practices. In small ruminants such as sheep (*Ovisaries*) and goats (*Capra hircus*), the utilization of alternative feeds, such as shrubs, plants, and agro-industrial by-products, can significantly enhance animal productivity, reduce carbon footprints, and mitigate greenhouse gas fluxes. This approach is environmentally friendly as it addresses critical issues associated with feed manufacturing, including land, energy, and water use, whilst also being cost-effective. Moreover, alternative feeds could be used to improve the quality of the meat and milk produced. By influencing the bacteria involved in ruminal biohydrogenation, these feeds can diminish methane emissions, thereby contributing to a reduction in the overall carbon footprint. 

## Data Availability

The manuscript includes all the necessary data, which are presented in the body of the text, tables, and figures.
